# A nomogram based on a patient-reported outcomes measure: predicting the risk of readmission for patients with chronic heart failure

**DOI:** 10.1186/s12955-020-01534-6

**Published:** 2020-08-27

**Authors:** Qiang Han, Jia Ren, Jing Tian, Hong Yang, Qing Zhang, Ruoya Wang, Jinghua Zhao, Linai Han, Chenhao Li, Jingjing Yan, Ke Wang, Chu Zheng, Qinghua Han, Yanbo Zhang

**Affiliations:** 1 Department of Health Statistics, School of Public Health, Shanxi Medical University, 56 South XinJian Road, Taiyuan, 030001 Shanxi Province China; 2 Shanxi Provincial Key Laboratory of Major Diseases Risk Assessment, 56 South XinJian Road, Taiyuan, 030001 Shanxi Province China; 3 Department of Cardiology, The 1st Hospital of Shanxi Medical University, 85 South Jiefang Road, Taiyuan, 030001 Shanxi Province China

**Keywords:** Patient-reported outcomes measure, Chronic heart failure, Readmission, Nomogram

## Abstract

**Background:**

Health-related quality of life, as evaluated by a patient-reported outcomes measure (PROM), is an important prognostic marker in patients with chronic heart failure. This study aimed to use PROM to establish an effective readmission nomogram for chronic heart failure.

**Methods:**

Using a PROM as a measurement tool, we conducted a readmission nomogram for chronic heart failure on a prospective observational study comprising of 454 patients with chronic heart failure hospitalized between May 2017 to January 2020. A Concordance index and calibration curve were used to evaluate the discriminative ability and predictive accuracy of the nomogram. A bootstrap resampling method was used for internal validation of results.

**Results:**

The median follow-up period in the study was 372 days. After a final COX regression analysis, the gender, income, health care, appetite-sleep, anxiety, depression, paranoia, support, and independence were identified and included in the nomogram. The nomogram showed moderate discrimination, with a concordance index of 0.737 (95% CI 0.673–0.800). The calibration curves for the probability of readmission for patients with chronic heart failure showed high consistency between the probability, as predicted, and the actual probability.

**Conclusions:**

This model offers a platform to assess the risk of readmission for different populations with CHF and can assist clinicians with personalized treatment recommendations.

## Introduction

Re-admission is the main adverse outcome for patients with heart failure (HF). Because it could be associated with a high mortality, lead to a decreased quality of life, and cause a significant financial burden [1–3]. Thus, it is important to assess the prognosis of HF, as patients at higher risk of poor outcomes could receive more intensive therapy and close monitoring [4]. There are already efforts to develop novel prognostic models for HF [5]. A number of clinical studies predicting hospitalization for the deterioration of HF have been summarized in detail by Rahimi et al. [6, 7] The majority of effective models were built based on clinical data collected from discharge records of patients [8]; however, research indicates that high health-related quality of life (HRQOL) predicts a more favorable prognosis in patients with cardiovascular disease [9]. Thus, HRQOL should be used as a new prognostic indicator for HF patients [10, 11], and as an independent predictive factor of readmission with HF [12, 13].

The patient-reported outcomes measure (PROM) evaluates a patient’s quality of life by the way of scales to promote communication, inspect and identify HRQOL issues, enhance patient-centered treatment, and increase patient satisfaction [14]. PROM is widely used in HRQOL and combines all aspects of emotional, psychological, physical, and social functions, and even includes an individual’s subjective perception of health [15, 16]. Over the years, there has been considerable progress in the measurement, analysis, and interpretation of PROM, which has been validated by multifarious studies [17]. Thus, PROM is helpful in diagnosis and therapy, and is of significant importance in clinical practice [18, 19] and widely recognized by medical professionals.

Nomograms have been regarded as dependable instruments by creating simple intuitive diagrams of the predictive models that quantify the risk of clinical adverse events [20, 21]. In recent years, nomograms have also been used to predict the prognosis of HF [22–24]. This study, as far as we know, is the first attempt to construct a prognostic nomogram of chronic heart failure (CHF) based on the Chinese PROM data of 454 patients with CHF, to predict the possibility of readmission.

## Methods

### Study design and sample

This study is a prospective observational study, from May 2017 to January 2020, patients in the department of cardiology of the top three hospitals in the Shanxi Province (the first affiliated hospital of Shanxi Medical University, Shanxi Cardiovascular Hospital and Bethune Hospital) who were consecutively chosen for the study. We conducted a questionnaire survey of inpatients who met the inclusion criteria and screened the filled scale. Follow-up calls were made via phone at 1, 3, 6, 12, 18, and 24 months after discharge to record whether a patient was readmitted, as well as the readmission time.

### Inclusion and exclusion criteria

Patients eligible for participation in the study had to meet the following criteria: (1) aged ≥18 years, (2) diagnosed with HF, according to the guideline for the diagnosis and treatment of HF in China (2018) [25], (3) fall under the New York Heart Association (NYHA) functional class II-IV, and (4) received HF treatment while in the hospital. Patients were excluded if they suffered from acute cardiovascular events within two months, or if they refused to participate in the project.

### Measures

#### CHF-prom

The study uses a Chinese questionnaire related to mainland China – CHF-PROM [26]. The CHF-PROM is divided into four domains which are further divided into 12 subdomains, including a total of 57 items. The structural frame of CHF-PROM is shown in Table 1.

**Table 1:** Structure of CHF-PROM

Domains	Dimensions	Variables	Num of items	Items
Physiology	Somatization	SOM	8	PHY1-、PHY2-、 ……、PHY7-、PHY8-
	Appetite Sleep	APS	4	PHY9-、PHY10-、PHY11-、PHY12-
	Independence	IND	4	PHY13、PHY14、PHY15、PHY16
Psychology	Anxiety	ANX	8	PSY1-、PSY2-、……PSY7-、PSY8-
	Depression	DEP	6	PSY9-、PSY10-、……、PSY13-、PSY14-
	Fear	FEA	3	PSY15-、PSY16-、PSY17-
	Paranoia	PAR	4	PSY18-、PSY19-、PSY20-、PSY21-
Society	Support	SUP	5	SOY1、SOY2、SOY3、SOY4、SOY5
	Utilization	UTI	3	SOY6、SOY7、SOY8
Treatment	Compliance	COM	2	TRE1、TRE2
	Satisfaction	SAT	8	TRE3、TRE4、……、TRE9、TRE10
	Effect of drug	EOD	2	TRE11-、TRE12-

Note: “-” indicates reverse scoring

In this study, the reliability of 12 subdomains were considered acceptable: Cronbach’s alpha coefficient > 0.60, (i.e., SOM (0.691), APS (0.617), IND (0.846), ANX (0.750), DEP (0.823), FEA (0.787), PAR (0.884), SUP (0.726), UTI (0.809), COM (0.854), SAT (0.826), EOD(0.751)).

The CHF-PROM uses a 5-point Likert scale to rate the responses and the scores for each item range from 0 (never) to 4 (always). In the study, all responses were converted in the following way to ensure consistency between the PROM and answers to the 56 items: positively scoring items were recorded as the raw score, and negatively scoring items were recorded as 4 minus the raw score. This ensured that the score for each item still ranged from 0 to 4 and further, the correspondence of a higher score to a more positive PROM.

### Procedures

This study was approved by the ethics committee of Shanxi Medical University and we obtained the consent of all participants. We screened inpatients who met the diagnostic criteria from the hospital medical records system, and eligible patients were contacted and recruited through a face-to-face interview. Eligible patients who agreed to participate in the study filled out a questionnaire through which we obtained other clinical data, demographic data, as well as CHF-PROM data.

### Statistical analysis

Categorical variables in demographic characteristics were described by frequency, and the Log-Rank test was performed for comparison between groups. The median follow-up time was calculated by the Reverse Kaplan-Meier method. The variables of *P* < 0.05 and subdomains of PRO (patient-reported outcomes) were initially included in the Cox regression analysis to identify the factors that increase the risk of CHF readmission. A subset of the predictors was then selected using a step-forward method to obtain the final model that was relatively streamlined with the maximum concordance index (C-index) and minimal Akaike information criterion (AIC). The factors of the final regression model were included in the construction of the nomograms to assess readmission probability.

Discrimination and calibration were generally used to evaluate the performance of the nomogram [27]. The discrimination was evaluated by Harrell’s C-index, which is analogous to the area under curve (AUC), yet demonstrated to be more appropriate for censored data. The calibration curves can represent the calibration to appraise the consistency between predicted readmission and observed readmission. Moreover, 1000 bootstrap samples were used for internal validation to evaluate the performance of the prediction [28]. The missForest method used for imputing for the missing values. Double entry of data using Epidata3.1 software was done to control data quality. The Reverse Kaplan-Meier method were performed using SPSS version 22.0. Cox regression and nomogram were implemented by the Survival and Hmisc package in R 3.6.1. In all analyses, *P* < 0.05 was regarded as statistically significant.

## Results

### Baseline characteristics

A total of 978 completed CHF-PROMs were collected in this study, among which 210 were not followed up, 224 were followed up once, 241 were followed up twice, 195 were followed up three times, and 108 were followed up four times. 90 patients with NYHA class I were excluded. Finally, 454 patients who met the inclusion criteria and were followed up more than once were entered to construct and verify our prediction model. The median follow-up time in this study was 372 days. The characteristics of demography and clinic for the patients in the study cohort are listed in Table 2.

**Table 2:** Demographic characteristics of patients with CHF

	Without Readmission	Readmission	***P***
	(***n*** = 312)	(***n*** = 142)	
**Age > =70**	140 (46.2%)	79 (56.4%)	0.018
**Female**	119 (38.1%)	77 (54.2%)	0.004
**Manual workers**	165 (63.2%)	66 (53.7%)	0.153
**literacy**			0.225
Low	103 (34.1%)	46 (33.3%)	
Middle	160 (53.0%)	65 (47.1%)	
High	39 (12.9%)	27 (19.6%)	
**Health care**			0.043
City health care	188 (60.3%)	105 (73.9%)	
Rural health care	116 (37.2%)	35 (24.6%)	
Self- paying	8 (2.6%)	2 (1.4%)	
**Family history**	99 (33.3%)	40 (29.0%)	0.496
**Income**			0.003
Low	169 (54.2%)	57 (40.1%)	
Middle	139 (44.6%)	76 (53.5%)	
High	4 (1.3%)	9 (6.3%)	
**Smoking history**			0.042
No	157 (52.0%)	87 (64.4%)	
smoking cessation	75 (24.8%)	32 (23.7%)	
Yes	70 (23.2%)	16 (11.9%)	
**Drinking history**			0.083
No	199 (63.8%)	104 (73.2%)	
temperance	34 (10.9%)	16 (11.3%)	
Yes	79 (25.3%)	22 (15.5%)	
**NYHA**			0.535
II	119 (38.1%)	47 (33.1%)	
III	123 (39.4%)	66 (46.5%)	
IV	70 (22.4%)	29 (20.4%)	
**BMI**			0.109
< 18.5	23 (7.6%)	17 (12.5%)	
18.5 ~ 24.9	137 (45.4%)	72 (52.9%)	
≥ 25	142 (44.0%)	43 (36.8%)	

### Selected factors for the model

Demographic variables screened by univariate analysis (such as age, gender, smoking, health care, income) and PRO were selected into the multivariable analysis. The multivariable analysis analyses showed that the readmission of patients with CHF was significantly related with income (*P* = 0.049), appetite, sleep (*P* = 0.016), social support (*P* = 0.008), paranoia (*P* = 0.012) and independence in terms of self-care ability, as well as the performance of daily activities (*P* < 0.001). However, age, gender, health care, smoking, somatization, anxiety, depression, fear, satisfaction with hospital treatment services, and compliance were not significant. The final prediction model with maximal C-index and minimum AIC was obtained by the step-forward selection, which included gender, income, health care, appetite-sleep, anxiety, depression, paranoia, support, and independence (Table 3).

**Table 3:** Cox regression analysis of the risk of readmission for patients with CHF

	Multivariable analysis			Selected factors for model		
Variables	*HR*	95% *CI*	*P*	*HR*	95% *CI*	*P*
Age	0.829	(0.556,1.236)	0.358			
Female	0.829	(0.556,1.236)	0.358	1.349	(0.951,1.914)	0.093
Income						
low	Reference			Reference		
middle	1.482	(1.001,2.194)	0.049	1.285	(0.879,1.879)	0.195
high	2.331	(0.955,5.685)	0.063	2.454	(1.123,5.366)	0.024
Health care						
urban health care	Reference			Reference		
rural health care	0.736	(0.469,1.153)	0.180	0.745	(0.487,1.141)	0.175
self pay	0.279	(0.038,2.059)	0.211	0.401	(0.098,1.646)	0.205
Smoking	0.913	(0.684,1.220)	0.540			
Somatization	0.994	(0.960,1.029)	0.719			
Appetite Sleep	0.924	(0.867,0.986)	0.016	0.929	(0.876,0.986)	0.015
Anxiety	0.966	(0.928,1.006)	0.090	0.960	(0.925,0.996)	0.030
Depression	0.964	(0.913,1.018)	0.188	0.971	(0.926,1.017)	0.208
Paranoia	1.158	(1.032,1.298)	0.012	1.124	(1.016,1.242)	0.023
Fear	0.968	(0.870,1.078)	0.554			
Support	0.935	(0.889,0.983)	0.008	0.931	(0.893,0.971)	< 0.001
Utilization	0.988	(0.929,1.051)	0.707			
Independence	0.929	(0.890,0.969)	< 0.001	0.930	(0.896,0.966)	< 0.001
Satisfaction	0.986	(0.947,1.027)	0.505			
Compliance	1.016	(0.897,1.151)	0.799			
Effect of drug	0.980	(0.900,1.068)	0.651			

### Predictive Nomogram for the probability of readmission

**Fig. 1:**
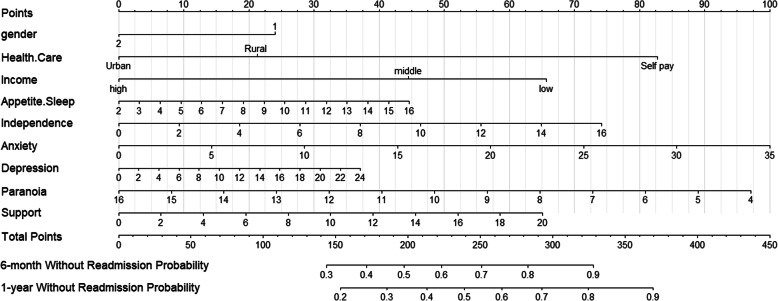
The nomogram predicting the risk of readmission for patients with CHF

Based on the final Cox regression analysis, the nomogram that including nine predictors of appetite-sleep, independence, anxiety, depression, paranoia, support, health care, gender, and income was built to estimate probability of without readmission (Fig. 1). The score of each variable was revealed on the points axis and the total score with seven predictors was reached by adding each point. Further, by putting the total points projection on the probability axis, we could directly calculate the probability of without readmission (Probability of readmission = 1 - Probability of without readmission). For example, for middle income males, enrolled in the rural health care program, with an appetite-sleep score of 5, independence score of 6, anxiety score of 5, depression score of 8, paranoia score of 12 and support score of 6, the total score calculated would be the sum of: 23.5 + 45 + 21 + 10 + 27.5 + 15 + 12.5 + 32.5 + 20 = 207. This corresponded with a 6-month without readmission probability of 0.54 and 1-year without readmission probability 0.38. Therefore, this person was approximately at 46% risk of readmission at 6-months and 62% risk of readmission at 1 year.

### Performance of the Nomogram

**Fig. 2:**
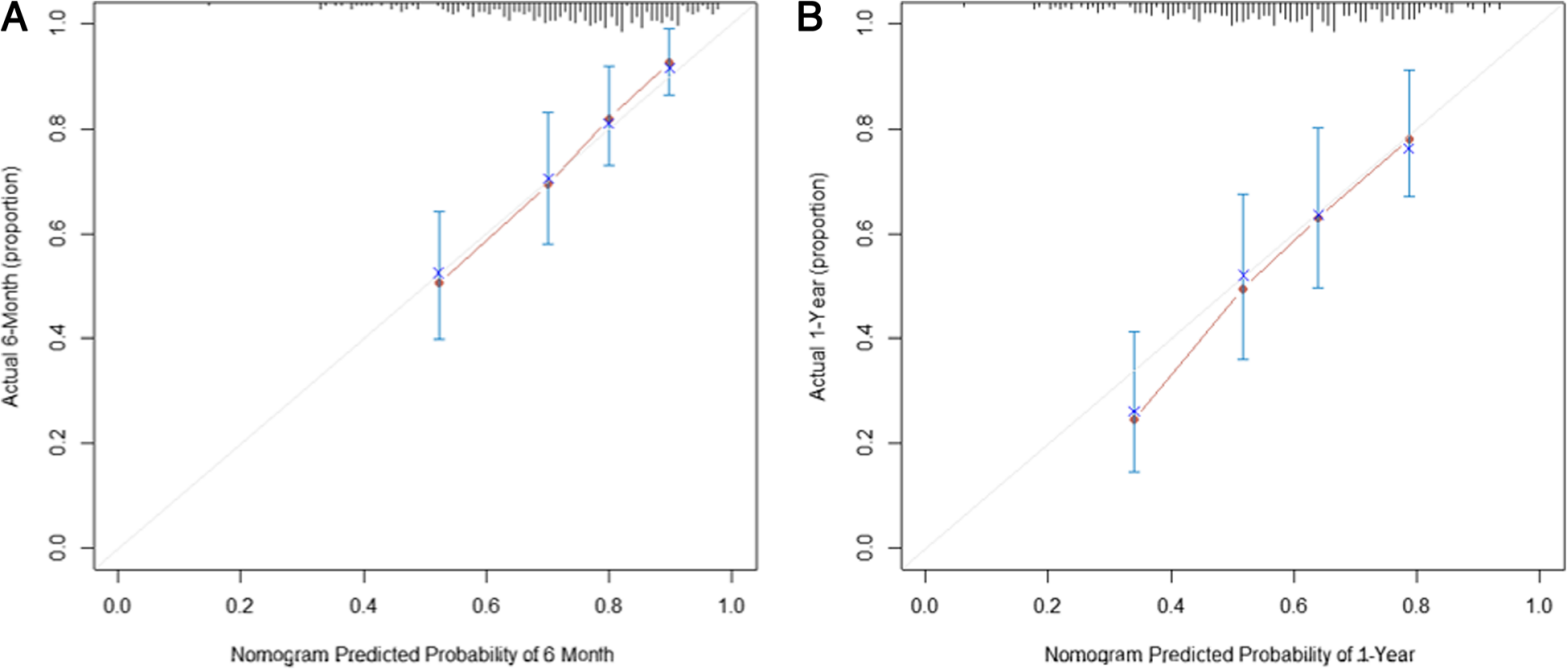
The calibration curve for predicting patients without readmission at (A) 6 months and (B) 1 year in the cohort. The x-axis represents the overall predicted probability of without readmission and the y-axis represents the actual probability. Model calibration is indicated by the degree of fitting of curve and the diagonal

The discrimination of the nomogram was measured by the C-index and the calibration was assessed using a calibration curve comparing the predicted probabilities and observed probabilities. The C-index value of 0.5 indicates no predictive discrimination and a value of 1.0 indicates the perfect separation of patients with different outcomes [21]. The C-index for a prognostic model is typically between about 0.6 and 0.85 (the larger the c-index, the more accurate the prognostic prediction) [29]. Calibration can be investigated by plotting the observed proportions of events against the predicted risks for groups defined by ranges of individual predicted risks. Ideally, if the observed proportions of events and predicted probabilities agree over the whole range of probabilities, the plot shows a 45° line (that is, the slope is 1) [29]. Bootstraps with 1000 resample were used for these activities. The nomogram displayed moderate discrimination with a C-index of 0.737 (95% CI 0.673–0.800) and splendid calibration. The calibration curve of the accuracy estimated by bootstrap (1000 resampling) was highly consistent with the diagonal, indicating that the predicted probability of ‘without readmission’ was in accordance with the actual probability (Fig. 2).

## Discussion

Many studies show that HRQOL is independent predictive factors of prognosis for inpatients with HF and that they have important predictive value [12, 13] and nomogram based on Cox regression has been widely used to predict the survival time of chronic diseases, especially cancer [30–32]. However, as far as we know, there have been no studies based on it to construct the quantitative model to predict the probability of readmission for patients with CHF. This study constructed a simple intuitive graph of the prediction model based on PROM to quantify the risk of readmission for CHF. This can be an important aid when doctors make treatment recommendations for patients with CHF.

There are some things that may be highlighted in this study. First, PROM used in our study is a questionnaire in Chinese based on the different cultural and societal value systems of mainland China as well as the medical and economic environments of the country. The reliability and validity of the scale have been verified by Tian et al. [26], and they were further verified and screened in this study. Second, only patients with CHF were selected in the study, regardless of etiology, LVEF, complications, etc. Thus, the database had covered, and was representative of, a wider population, further promoting the clinical application of the model. Third, internal validation through a bootstrap resampling method demonstrated moderate discrimination and excellent calibration, illustrating that the nomogram based on PROM may be valuable for patients with CHF.

This study, using data from strict screening and regular follow-up of CHF patients, confirms the significance of some demographic characteristics for prognosis; the results are consistent with those from other studies [33–36]. In our prediction model, anxiety showed the greatest effect on the risk of readmission, followed by paranoia, health care, independence, income, support and appetite-sleep, while the smallest contributors were gender and depression.

Recently, a prospective observational study provided evidence of physical weakness, independence, support from society and family, anxiety, and depression being likely predictors of 30-day prognosis after hospitalization for HF [37]. Staniute et al. further also demonstrated that anxiety, depression and social support can indirectly affect the quality of life of patients with HF [38]. Moreover, anxiety, appetite and sleep were confirmed to be predictors of readmission for CHF in retrospective studies, which may have been impacted by the fact that the patient’s status influences the risk of readmission [39–42]. Kitamura M not only confirmed that daily activities were independent predictors of readmission in heart failure patients within 90 days, but also calculated the cut-off value by ROC curve [43] and the study have also proved that self-care and daily activities are the mediating factors of readmission of heart failure [44]. While Hochang Benjamin Lee et al. emphasized that personality disorders as predictors of incident cardiovascular disease increased risk disease [45], our results are quite the opposite, which may be because the subjects studied were different; Apart from the results on patient paranoia, these findings were similar to the results of our reports on the readmission risk factors for CHF. We found that gender, income, health care, appetite-sleep, anxiety, depression, paranoia, support, and independence were predictors of readmission for CHF.

Many clinicians are usually able to make a preliminary assessment of a patient’s prognosis through clinical data; however, combining it with PRO can provide a more comprehensive and accurate understanding of the real health status of the patient. In practice, the process has been streamlined as the simple nomogram can be incorporated in mobile applications.

It would also be important to note some limitations of the study. First, according to the analysis, patients who refuse to follow up may be worse off than those who cooperate with follow-up, so that excluding these patients will underestimate the rehospitalization rate of patients with chronic heart failure. In addition, because the scale was subjectively filled by the patient, part of the content in the scale may be missing. Although we have imputed the missing data, there may still be bias. Second, most critical patients were not included in the study due to their inability to complete the scale. Due to this selection bias, our model may underestimate patient readmission rates. If all patients who meet the inclusion criteria could be selected, the actual C-index might be higher. Third, though the internally validated model demonstrated moderate discrimination and splendid calibration, considering the epidemiological and clinical behavioral differences between regions, the universality of this nomogram still requires additional databases to be used for external validation, especially from other provinces.

## Conclusion

In conclusion, by using PROM as a measurement tool, this study has constructed and internally validated a neoteric nomogram to predict the probability of readmission for CHF patients. The nomogram is moderate accurate, easy to use, and displays splendid calibration; so, it can assist clinicians in assessing the risk of readmission for CHF patients, and further, make personalized treatment recommendations for them.

## Data Availability

Please contact the corresponding author for the study data, which will be granted upon reasonable request.

## Abbreviations

CHF-PROM: Patient-reported outcomes measure for chronic heart failure
HRQoL: Health-related quality of life
C-index: Concordance index
AIC: Akaike information criterion
C. I: Confidence interval
RR: Relative risk
SE: Standard error
